# Emergence of Recombinant Forms of HIV: Dynamics and Scaling

**DOI:** 10.1371/journal.pcbi.0030205

**Published:** 2007-10-26

**Authors:** Gajendra W Suryavanshi, Narendra M Dixit

**Affiliations:** Department of Chemical Engineering, Indian Institute of Science, Bangalore, India; Swiss Federal Institute of Technology Zurich, Switzerland

## Abstract

The ability to accelerate the accumulation of favorable combinations of mutations renders recombination a potent force underlying the emergence of forms of HIV that escape multi-drug therapy and specific host immune responses. We present a mathematical model that describes the dynamics of the emergence of recombinant forms of HIV following infection with diverse viral genomes. Mimicking recent in vitro experiments, we consider target cells simultaneously exposed to two distinct, homozygous viral populations and construct dynamical equations that predict the time evolution of populations of uninfected, singly infected, and doubly infected cells, and homozygous, heterozygous, and recombinant viruses. Model predictions capture several recent experimental observations quantitatively and provide insights into the role of recombination in HIV dynamics. From analyses of data from single-round infection experiments with our description of the probability with which recombination accumulates distinct mutations present on the two genomic strands in a virion, we estimate that ∼8 recombinational strand transfer events occur on average (95% confidence interval: 6–10) during reverse transcription of HIV in T cells. Model predictions of virus and cell dynamics describe the time evolution and the relative prevalence of various infected cell subpopulations following the onset of infection observed experimentally. Remarkably, model predictions are in quantitative agreement with the experimental scaling relationship that the percentage of cells infected with recombinant genomes is proportional to the percentage of cells coinfected with the two genomes employed at the onset of infection. Our model thus presents an accurate description of the influence of recombination on HIV dynamics in vitro. When distinctions between different viral genomes are ignored, our model reduces to the standard model of viral dynamics, which successfully predicts viral load changes in HIV patients undergoing therapy. Our model may thus serve as a useful framework to predict the emergence of multi-drug-resistant forms of HIV in infected individuals.

## Author Summary

Retroviral recombination, a process akin to sexual reproduction in higher organisms, may accelerate the accumulation of mutations and the development of multi-drug resistance in HIV patients. Recombination occurs when the enzyme reverse transcriptase switches between the two RNA strands of a virion, yielding a provirus that is a mosaic of the two strands. The latter strands are often distinct, thereby allowing recombinational diversification, when multiple viruses infect individual cells. The enormous HIV recombination rate and recent evidence of frequent multiple infections of cells render recombination a powerful force underlying the development of multi-drug resistance in vivo. The dynamics of the emergence of recombinant genomes, however, remains poorly understood. Recent experiments allow a closer look at HIV recombination: cells are exposed to two kinds of reporter viruses and the frequency of recombinant proviruses is detected, which enables direct quantification of the extent of recombination. The observations, however, are not described by available models, leaving a gap in our understanding of HIV recombination. We present a model that describes HIV dynamics with multiple infections of cells and recombination, captures several recent experimental observations quantitatively, provides insights into HIV recombination, and presents a framework for describing the development of multi-drug resistance in HIV patients.

## Introduction

During the reverse transcription of HIV in an infected cell, the viral enzyme reverse transcriptase switches templates frequently from one genomic RNA strand of a virion to the other, yielding a recombinant proviral DNA that is a mosaic of the two parent genomes. If one strand contains a mutation that confers upon HIV resistance to one administered drug and the other strand resistance to another drug, recombination may bring the two mutations together and give rise to progeny genomes resistant to both those drugs [1,2]. Recombination may thus accelerate the emergence of multi-drug resistance in infected individuals. A prerequisite for recombination to induce genomic diversification is the presence of heterozygous virions [3], which contain nonidentical genomic RNA strands and are formed when individual cells are infected by multiple virions. Recent experiments present evidence of the predominance of multiple infections of cells both in vitro and in vivo [4–7]: infected splenocytes from two HIV patients, for instance, were found to harbor up to eight proviruses, with three to four proviruses per cell on average [6]. The high incidence of multiple infections of cells coupled with the high recombination rate of HIV, estimated to be several times greater than the HIV point mutation rate [7–10], sets the stage for recombination to act as a powerful agent driving the emergence of multi-drug-resistant forms of HIV in patients undergoing therapy. In addition, recombination may serve to preserve diversity in genomic regions not affected by bottlenecks introduced by drug therapy or host immune responses, and improve the adaptability of HIV to new environments [11]. Indeed, in addition to several circulating recombinant forms of HIV, a large number of recombinant forms unique to individuals have been identified [12]. It is of great importance, therefore, to understand how recombinant forms of HIV arise in infected individuals.

Remarkable insights into HIV recombination emerge from recent in vitro experiments, in which target cells were simultaneously exposed to two kinds of reporter viruses, and cells infected with recombinant proviruses detected [3–5,7,13,14]. Rhodes et al. determined using single-round infection assays that the likelihood of the accumulation by recombination of distinct mutations present on the two viral genomes in a virion increases with the separation between the mutations and reaches an asymptotic maximum at a separation of ∼1,500 base pairs [14]. Further, Rhodes et al. found that the cell type employed for infection—CD4^+^ T cells or macrophages, for instance—does not influence the recombination rate. In contrast, Levy et al. argue that subtle virus–cell interactions cause recombination to occur at different rates in different types of cells [7]. Further, using replication-competent reporter viruses, Levy et al. investigated the dynamics of the emergence of recombinant genomes in vitro and in SCID-hu mice [7]. Interestingly, Levy et al. observed two scaling patterns. First, the percentage of cells coinfected with both the reporter genomes employed for infection was proportional to the total percentage of infected cells. Second, the percentage of cells infected with recombinant proviruses was linearly proportional to the percentage of coninfected cells and, correspondingly, proportional to the square of the total percentage of infected cells. Further, Levy et al. found that the scaling patterns were independent of the initial viral load, the time following the onset of infection, and whether the experiments were conducted in vitro or in SCID-hu mice.

Standard models of viral dynamics, which successfully describe short-term (a few weeks) viral load changes in patients undergoing therapy, are predicated on the infection of individual cells by single virions and ignore recombination [15–17]. Recent modeling advances and simulations incorporate descriptions of multiple infections of cells and recombination, and present insights into the subtle interplay between mutation, recombination, fitness selection, and random genetic drift that underlies the genomic diversification of HIV in vivo [18–23]. Bretscher et al. developed a model of HIV dynamics that includes mutation, double infections of cells, and recombination and found that for infinitely large cell populations, the influence of recombination on the development of drug resistance depends sensitively on epistasis, i.e., on the nature of fitness interactions between mutations [20]. Bretscher et al. argue that phenotypic mixing—the assortment of viral proteins arising from different proviral genomes within a multiply infected cell during the assembly of progeny virions—compromises the selective advantage of the fittest strains and enhances the relative abundance of less fit strains: less fit strains piggyback on fitter strains. At the same time, in a two-locus/two-allele model, recombination, which breaks nonrandom associations of mutations and hence lowers linkage disequilibrium, enhances the relative abundance of single mutant strains compared to wild-type and of double mutant strains when fitness interactions result in positive epistasis, i.e., when mutations interact antagonistically in lowering viral fitness. Bretscher et al. predict, contrary to the prevalent paradigm, that phenotypic mixing and positive epistasis together result in a deceleration of the growth of drug-resistant viruses upon increasing the recombination rate [20]. Recent experimental evidence points to a mean positive epistasis underlying fitness interactions in HIV-1, which, following the predictions of Bretscher et al. [20], raises questions about the benefits of recombination to HIV-1 and, more generally, of the evolutionary origins of recombination and sexual reproduction [24].

Fraser presents a detailed model of HIV dynamics considering up to three infections of cells, mutation, recombination, fitness selection, and different dependencies of the frequency of multiple infections of cells on the viral load [22]. In agreement with Bretscher et al. [20], Fraser found that recombination inhibits the development of drug resistance during antiretroviral therapy and, further, that this effect is modulated not only by epistasis but also by the dependence of the frequency of multiple infections on viral load [22].

In a more recent study, Althaus and Bonhoeffer extend the description of Bretscher et al. [20] to finite population sizes, where the relative abundance of different mutant strains may be determined stochastically rather than deterministically [18]. Interestingly, Althaus and Bonhoeffer found that even when positive epistasis governs fitness interactions between resistance mutations, recombination may significantly accelerate the development of drug resistance when the effective population size is ∼10^4^–10^5^ [18]. Using bit-string simulations, Bocharov et al. found that multiple infections of cells and recombination act in synergy to enhance viral genomic diversity [19], which in turn may increase the likelihood of the emergence of drug resistance. Bocharov et al. note, however, that the time for the selection of fitter genomes that contain multiple mutations may be highly variable, indicative of the stochastic nature of viral evolution in vivo [19]. Rouzine and Coffin developed a description of viral evolution with fitness selection, recombination between multiple loci, and random genetic drift, and predict that below a critical population size, the viral population within an individual may converge to a clone in the absence of mutation, leaving little scope for recombination to introduce genomic diversification [23]. Rouzine and Coffin suggest therefore that a reduction of the viral population in an infected individual by combination antiretroviral therapy may decelerate significantly the emergence of drug resistance [23]. The effective population size in vivo remains to be established [25].

Currently available models thus make valuable predictions of the influence of recombination on HIV dynamics and the emergence of drug resistance in infected individuals undergoing therapy. The predictions, however, are diverse and have not been compared with available experimental data [3–5,7,13,14]. An important gap thus exists in our understanding of HIV recombination. Consequently, for instance, the origins of the experimental scaling and dynamical patterns associated with HIV recombination [7] remain poorly understood. Similarly, the recombination rate, or the frequency of template switching events during reverse transcription, remains to be established [7,14].

One limitation of currently available models lies in the approximate descriptions of the dynamics of multiple infections of cells employed. For example, the frequency with which cells are doubly infected is assumed either to be constant [18,20], proportional to the viral load, or proportional to the square of the viral load [22]. Because multiple infections of cells are a prerequisite for the formation of heterozygous virions, an accurate description of HIV dynamics with recombination depends critically on the underlying description of multiple infections, as suggested also by Fraser [22]. The frequency of multiple infections depends not only on the viral load, but also on CD4 down-modulation induced by viral gene expression following the first infection of a cell [26–28], which lowers the susceptibility of the cell to further infections. In a recent study, Dixit and Perelson developed a model that explicitly accounts for CD4 down-modulation and presents a rigorous description of the orchestration of multiple infections of cells by free virions [29]. The model elucidates the origins of one of the two scaling relationships observed by Levy et al. [7]: that the number of doubly infected cells is proportional to the square of the total number of infected cells. Levy et al. note that at the onset of infection in their experiments, because equal numbers of the two kinds of reporter viruses are employed, the probability that a cell is infected with both the reporter genomes is the product of the probabilities that the cell is infected independently with each of the genomes, which explains the origin of the observed scaling early during infection. Later in the infection process, multiple infections of a cell need not be simultaneous and may be sequential. Yet, the quadratic scaling persists. Levy et al. speculate that the absence of any functional hindrance to multiple infections may underlie the persistence of the scaling throughout the infection period. The inhibition of multiple infections by CD4 down-modulation, however, may not be negligible. Dixit and Perelson consider the expected inhibition of multiple infections by CD4 down-modulation and identify conditions under which the observed scaling relationship may hold [29].

The latter model, however, does not distinguish between different viral genomes that infect cells and thereby precludes a description of recombination. Thus, for instance, the dynamics of the emergence of recombinant genomes and the origins of the second scaling relationship observed by Levy et al.—that the percentage of cells infected by recombinant genomes is linearly proportional to the percentage of cells coinfected with both the reporter genomes employed for infection—remain to be elucidated.

The analysis of Dixit and Perelson [29] provides insights into the origins of the latter scaling. According to the analysis, one set of conditions under which the quadratic scaling between the population of coinfected cells and that of all infected cells holds occurs at time periods that are long compared to the characteristic viral production and clearance times, so that viral populations are in pseudo equilibrium with the infected cell populations [29]. Under the same conditions, when two distinct reporter genomes are employed for infection, the population of heterozygous virions containing a copy each of the two reporter genomes is expected be in pseudo equilibrium with, and hence proportional to, the population of cells coinfected with both the reporter genomes. Further, for time periods long compared to the lifetimes of infected cells and the characteristic timescale of the infection of uninfected cells, the production and death rates of infected cells are expected to exhibit a pseudo steady state. The number of cells infected with recombinant proviruses would then be proportional to the population of heterozygous virions and hence to the population of coinfected cells, which may explain the origins of the second scaling relationship observed by Levy et al. [7]. (We present more mathematical arguments below.)

Whether currently available models of HIV dynamics that include infections by distinct viral genomes and recombination validate the above arguments and predict the observed scaling remains unclear. Dixit and Perelson predict the existence of the above scaling relationships under certain parameter regimes and, importantly, that the scaling relationships may depend on the length of time following the onset of infection [29]. In contrast, Levy et al. found that the scaling is independent of the time following the onset of infection [7]. Further, Levy et al. found that for the different initial viral loads employed, which varied well over two orders of magnitude, the parametric plots of different cell populations defining the scaling relationships superimpose remarkably tightly [7], whereas a similar superimposition at all times following the onset of infection is not apparent from the above scaling arguments, which hold after the establishment of pseudo equilibrium between viral production and clearance. A comprehensive model of HIV dynamics with recombination that quantitatively captures available experimental data is currently lacking.

In this work, we develop a detailed model of HIV dynamics that considers multiple infections of cells by distinct viral genomes and describes recombination. Our model captures several recent in vitro experimental findings quantitatively and provides key insights into the mechanisms underlying the emergence of recombinant forms of HIV. At the same time, our model is consistent with the standard model of viral dynamics, which successfully captures viral load changes in patients following the onset of antiretroviral therapy, and may therefore be extended to describe HIV dynamics with recombination in vivo.

## Results

### Model Formulation

**Figure 1:**
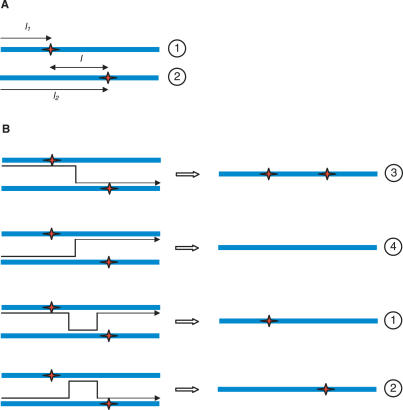
Schematic Representation of Viral Genomes and Recombination Viral genomes 1 and 2 employed at the onset of infection (A) and the four genomes resulting from the recombination of genomes 1 and 2 (B).

We consider in vitro experiments where a population of uninfected CD4^+^ cells, *T*, is exposed simultaneously to two populations, *V*_11_ and *V*_22_, of homozygous virions containing genomes 1 and 2, respectively. The genomes 1 and 2 are assumed to be distinct at two nucleotide positions, *l*_1_ and *l*_2_, a distance *l* apart on the viral genome, with genome 1 having a mutation at position *l*_1_ and genome 2 at *l*_2_ (Figure 1A). Following exposure, target cells become infected singly or multiply with one of the genomes, or coinfected with both genomes. Coinfected cells produce heterozygous virions, *V*_12_, which contain a copy each of genomes 1 and 2.

Infection of target cells by the virions *V*_12_ yields two kinds of “recombinant” genomes depending on the template switching events during reverse transcription (Figure 1B). When the mutations at positions *l*_1_ and *l*_2_ are both included in the resulting proviral DNA, a recombinant genome that we denote as genome 3 is formed. When both the mutations are excluded, the other recombinant, genome 4, results. When one of the two mutations is included but not the other, genomes 1 and 2 are recovered. Thus, four kinds of viral genomes, 1, 2, 3, and 4, eventually infect cells.

We distinguish infected cells by the proviral genomes they contain. We denote by *T_i_* cells containing a single provirus *i*, where *i* ∈ {1, 2, 3, 4} represents the four genomes above. Thus, cells *T*_1_ contain a single provirus 1, cells *T*_2_ contain a single provirus 2, and so on. We denote by *T_ij_*, where *i* and *j* ∈ {1, 2, 3, 4}, cells that contain two proviruses. Thus, cells *T*_11_ contain two copies of provirus 1, and cells *T*_12_ contain a copy of provirus 1 and a copy of provirus 2. Because cells *T_ij_* are indistinguishable from cells *T_ji_*, we subject *i* and *j* to the constraint *i* ≤ *j*, resulting in ten kinds of doubly infected cells: *T*_11_, *T*_12_, *T*_13_, *T*_14_, *T*_22_, *T*_23_, *T*_24_, *T*_33_, *T*_34_, and *T*_44_. Extending the description, cells *T_ijk_* are infected with three proviruses, and so on. Our aim is to describe the dynamics of recombination observed in experiments that employ two kinds of reporter viruses to infect cells. In these experiments, the number of cells infected with more than two genomes is estimated to be small [7]. Therefore, and for simplicity, we restrict our model to single and double infections of cells.

Random assortment of viral RNA produced in infected cells gives rise to ten different viral populations, which we denote *V_ij_*, where *i* ≤ *j* and *i* and *j* ∈ {1, 2, 3, 4}, based on the viral genomes, *i* and *j*, contained in the virions. Thus, for instance, virions *V*_34_ contain a copy each of genomes 3 and 4. Cells infected with a single kind of provirus, *T_i_* and *T_ii_*, give rise to homozygous virions, *V_ii_*. Cells coinfected with distinct proviruses, *T_ij_*, produce the homozygous virions *V_ii_* and *V_jj_* and the heterozygous virions *V_ij_*.

Below, we write equations to describe changes in the various cell and viral populations following the onset of infection.

### Dynamical Equations

#### Uninfected cells.

The in vitro dynamics of the uninfected cell population is governed by [29]

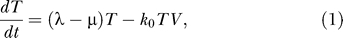
where *λ* and *μ* are the proliferation and death rates of uninfected cells in vitro, *k*_0_ is the second-order infection rate of uninfected cells, and 
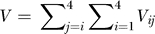
 is the total viral load. Equation 1 is constrained by the initial condition that the uninfected cell population at the onset of infection (*t* = 0) is *T*_0_.

#### Infected cells.

The singly infected cell subpopulations are determined by the integral equations:

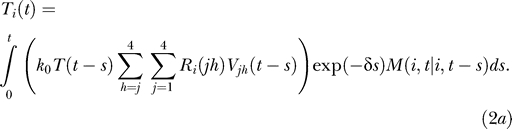
Here, *k*_0_ *T*(*t − s*)*V_jh_*(*t − s*)*ds* is the number of uninfected cells that are first infected by virions *V_jh_* in an infinitesimal interval of time *ds* near time *t − s* ≥ 0, where *t* = 0 marks the onset of infection. We define *R_i_*(*jh*) as the probability that provirus *i* results from the recombination of genomes *j* and *h*, where *i*, *j*, and *h* ∈ {1, 2, 3, 4} and *j* ≤ *h*. Thus, *k*_0_ *T*(*t − s*)*R_i_*(*jh*)*V_jh_*(*t − s*)*ds* is the expected number of uninfected cells first infected by virions *V_jh_* in the interval *ds* near time *t − s* and in which recombination results in provirus *i*. We assume that reverse transcription occurs rapidly following infection. Summation of *k*_0_ *T*(*t − s*)*R_i_*(*jh*)*V_jh_*(*t − s*)*ds* over *j* and *h* therefore yields the total number of uninfected cells that are first infected with a single provirus *i* in the interval *ds* near *t − s*. The probability that these latter cells survive until time *t* is exp(−*δs*), where *δ* is the death rate of infected cells. We define *M*(*i*,*t* | *i*,*t − s*) as the probability that a cell that is first infected with provirus *i* at time *t − s* remains singly infected with the provirus *i* at time *t* given that the cell survives the intervening interval of duration *s*. The integrand in Equation 2a thus represents the number of uninfected cells that are first infected in the infinitesimal interval *ds* near time *t − s* and survive with a single provirus *i* at time *t*. Integration over *s* from 0 to *t* gives the total number of cells containing a single provirus *i* at time *t*.

The doubly infected cell subpopulations are determined in an analogous manner:

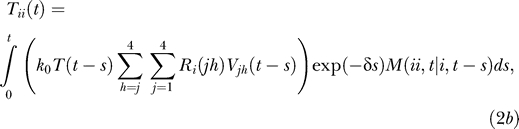
where *M*(*ii*,*t* | *i*,*t − s*) is the probability that a cell that is first infected with provirus *i* at time *t − s* contains an additional provirus *i* at time *t* given that the cell survives the intervening interval of duration *s*.

For cells coinfected with two different kinds of proviruses, we write

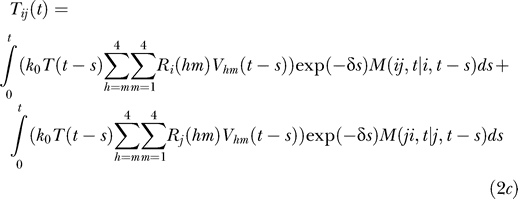
where *i* ≠ *j* and *M*(*ij*,*t* | *i*,*t − s*) is the probability that a cell that is first infected with provirus *i* at time *t − s* contains an additional provirus *j* at time *t* given that the cell survives the intervening interval of duration *s*. The two integrals in Equation 2c correspond to the two ways of acquiring the two proviruses: *i* followed by *j* and *j* followed by *i*, respectively.

#### Multiple infections.

To evaluate the conditional probabilities *M*, which characterize multiple infections, we consider a cell first infected with provirus *i* at time *t − s*. For times *τ* > *t − s*, the rate of infection of the cell reduces exponentially because of CD4 down-modulation [28], so that [29]

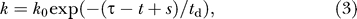
where *k*_0_ is the infection rate of an uninfected cell and *t*_d_ is the timescale of CD4 down-modulation. Three viral genes, *nef*, *env*, and *vpu*, acting via independent pathways, together induce nearly 100% down-modulation of CD4 molecules from the surface of an infected cell [26]. Of the three genes, the predominant influence is by *nef* [26], which induces rapid down-modulation of CD4 receptors following infection [28]. The latter down-modulation profile is well-described by an exponential decline (of timescale *t*_d_) and is extended to include the influence of *env* and *vpu* [29]. How the susceptibility of a cell to new infections declines with CD4 down-modulation remains unknown. Here, we follow Dixit and Perelson [29] and assume that the infection rate *k* is directly proportional to the CD4 expression level and hence declines exponentially with time following the first infection.

Assuming that the cell, following its first infection with provirus *i* at time *t − s*, does not die, the probability that it contains the provirus *i* alone at time *τ* is by definition *M*(*i*,*τ* | *i*,*t − s*). In a subsequent infinitesimal interval Δ*τ*, in which at most one infection may occur, the probability that the cell is not infected is (1 – *kV*(*τ*)Δ*τ*), where *k* is given by Equation 3 and 
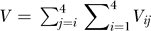
 is the total viral load. The probability that the cell remains singly infected with provirus *i* at time *τ* + Δ*τ* is therefore

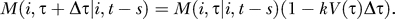
Subtracting *M*(*i*,*τ* | *i*,*t − s*), dividing by Δ*τ*, and letting Δ*τ* → 0, we obtain

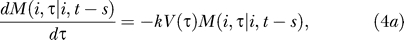
with the initial condition that *M*(*i*,*t* − *s* | *i*,*t* − *s*) = 1.

 Alternatively, a cell first infected with provirus *i* at time *t − s* may contain two proviruses, *i* and *j*, at time *τ +* Δ*τ* if it contains the provirus *i* alone at time *τ* and acquires an additional provirus *j* in the interval Δ*τ*, or if it contains both the proviruses *i* and *j* at time *τ*. (We ignore more than two infections of cells.) In the interval Δ*τ*, the probability that the cell acquires a second provirus *j* is 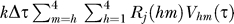 (see above), so that

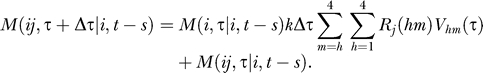
Subtracting *M*(*ij*,*τ* | *i*,*t − s*), dividing by Δ*τ*, and letting Δ*τ* → 0, we obtain

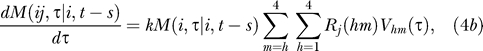
with the initial condition *M*(*ij*,*t* − *s* | *i*,*t* − *s*) = 0.

Substituting *j* by *i* in Equation 4b yields the corresponding evolution equation for *M*(*ii*,*τ* | *i*,*t* − *s*) with the initial condition *M*(*ii*,*t* − *s* | *i*,*t* − *s*) = 0.

#### Recombination.

We next determine the probability *R_i_*(*jh*) that provirus *i* results from the recombination of genomes *j* and *h*. For homozygous virions, *V_ii_*, reverse transcription yields the genome *i* alone so that

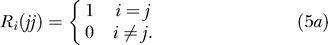
(Note that we ignore mutations here.) For heterozygous virions, we consider first those combinations where the two genomes *j* and *h* differ in a single position, which happens when *j* is either 1 or 2 and *h* is either 3 or 4 (Figure 1B). Because the difference is in a single position, reverse transcription yields either of the two genomes with equal probability. Thus,

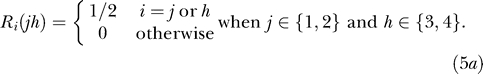



Finally, when *j =* 1 and *h =* 2 and when *j =* 3 and *h =* 4, the two genomes differ in two positions; we consider these combinations explicitly. Let *j =* 1 and *h =* 2. Recall that genome 1 has a mutation at position *l*_1_ and genome 2 at *l*_2_ and that *l*_2_ – *l*_1_ = *l* (Figure 1A). Recombination between genomes 1 and 2 yields genome 1 if the mutation on genome 1 is included in the resulting provirus and that on genome 2 is excluded (Figure 1B). Because reverse transcription is equally likely to begin on either genome, the probability that reverse transcriptase is on genome 1 at the position *l*_1_, i.e., the probability that the mutation on genome 1 is included in the resulting provirus, is 1/2. Given that the mutation on 1 is included, the mutation on 2 will be excluded if an even number of crossovers occurs between *l*_1_ and *l*_2_.

Let *n* be the average number of crossovers during reverse transcription of the viral genome of length *L*. We define the crossover frequency, or the recombination rate, as *ρ* = *n*/*L* crossovers per position. Assuming that crossovers occur independently, the probability *P*(*x*) that *x* crossovers occur in a length *l* of the genome follows the Poisson distribution [14,30]

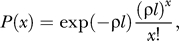
where *x*! = *x*(*x −* 1)...2.1. The probability that an even number of crossovers occurs in the length *l* is therefore the sum

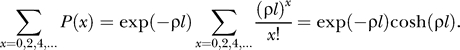
Thus, the probability that genome 1 results from recombination of genomes 1 and 2 is

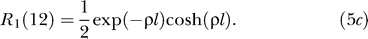



Similarly, genome 2 results from genomes 1 and 2 if the mutation on genome 1 is excluded and an even number of crossovers occurs between *l*_1_ and *l*_2_ so that the mutation on 2 is included. It follows that *R*_2_(12) = *R*_1_(12).

Genome 3 results from genomes 1 and 2 if the mutation on genome 1 is included and an odd number of crossovers occurs between *l*_1_ and *l*_2_ so that the mutation on genome 2 is also included. Following the above arguments, we find that

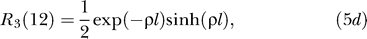
and that *R*_4_(12) = *R*_3_(12).

Similarly, when *j =* 3 and *h =* 4, we derive

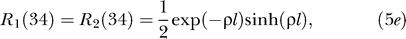
and

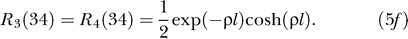



#### Virions.

Finally, we write equations for the time evolution of the various viral populations:

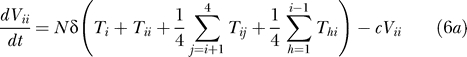
and

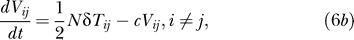
where we recognize that cells *T_i_* and *T_ii_* produce homozygous virions *V_ii_*, and cells *T_ij_* produce homozygous virions *V_ii_* and *V_jj_* and heterozygous virions *V_ij_* in the proportions 1/4, 1/4, and 1/2, respectively. *N* is the viral burst size and *δ* is the death rate of infected cells, both assumed to be independent of the multiplicity of infection [29]. Equations 6a and 6b are constrained by the initial condition that the viral population at the onset of infection is composed of equal subpopulations of the homozygous virions *V*_11_ and *V*_22_ alone, i.e., *V*_11_ = *V*_22_ = *V*_0_ at *t =* 0.

Equations 1–6 present a model of HIV dynamics with multiple infections of cells and recombination.

### Model Predictions

We solve Equations 1–6 (Methods) using the following parameter estimates drawn from in vitro studies [29,31]: the birth and death rate of target cells, *λ* = 0.624 d^−1^ and *μ* = 0.018 d^−1^; the death rate of infected cells, *δ* = 1.44 d^−1^; the viral burst size, *N* = 1,000; and the clearance rate of free virions, *c* = 0.35 d^−1^. We let an initial target cell population, *T*_0_ = 10^6^, be exposed to two equal viral populations, *V*_11_ = *V*_22_ = *V*_0_, which we vary over the experimental range, 2*V*_0_ = 10^6^ to 10^10^ [7]. The infection rate constant, *k*_0_, the timescale of CD4 down-modulation, *t*_d_, and the recombination rate, *ρ*, are not well-established, and we vary these parameters over ranges that define their best current estimates. We choose *l*, the separation between the mutations on genomes 1 and 2, in accordance with experiments (see below).

#### Virus and cell dynamics.

**Figure 2:**
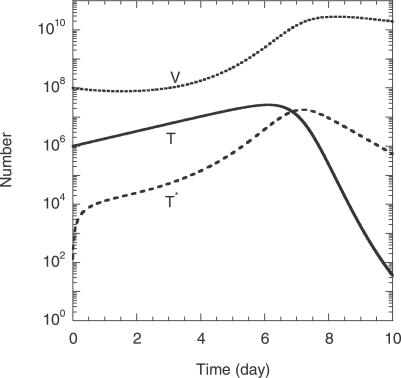
Model Predictions of the Overall Cell and Viral Dynamics The time evolution of the number of uninfected cells, *T*, the total number of infected cells, T^*^, and the total viral load, *V*, following the onset of infection obtained by the solution of Equations 1–6 with the following parameter values: the initial target cell number, *T*_0_ = 10^6^; the initial viral load, 2*V*_0_ = 10^8^; the birth and death rates of uninfected cells, *λ* = 0.624 d^−1^ and *μ* = 0.018 d^−1^; the death rate of infected cells, *δ* = 1.44 d^−1^; the viral burst size, *N* = 1,000; the clearance rate of free virions, *c* = 0.35 d^−1^; the infection rate constant of uninfected cells, *k*_0_ = 2 × 10^−10^ d^−1^; the CD4 down-modulation timescale, *t*_d_ = 0.28 d; the recombination rate, *ρ* = 8.3 × 10^−4^ crossovers per position; and the separation between the mutations on genomes 1 and 2, *l* = 408 base pairs.

In Figure 2, we present the time evolution of uninfected cells, *T*, the total infected cell population, 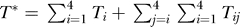 , and the total viral load, 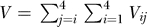 , for the parameter values 2*V*_0_ = 10^8^, *k*_0_ = 2 × 10^−10^ d^−1^, *t*_d_ = 0.28 d, ρ = 8.3 × 10^−4^ crossovers per position (see below), and *l* = 408 base pairs. We find that *T*, T^*^, and *V* evolve in two dominant phases, an initial rise and a subsequent fall. The initial rise in *T* is due to the net proliferation of uninfected cells at the rate (*λ−μ*)*T* (Equation 1), which in the initial stages of infection is large compared to the loss of uninfected cells by infection at the rate *k*_0_ *VT*. The latter infection process causes T^*^ to rise. The rise in T^*^ and hence viral production results in an increase in *V*. When *V* becomes large, the loss of uninfected cells by infection dominates cell proliferation and induces a decline in *T*. In Figure 2, *T* reaches a maximum at time *t* ≈ 6 d after the onset of infection. The decline in *T* lowers the formation of infected cells and T^*^ decreases at the death rate *δ*. Finally, the loss of T^*^ lowers viral production and induces a decline in *V* at the clearance rate *c*. This overall two-phase dynamics is similar to the T cell dynamics observed in vitro [7].

**Figure 3:**
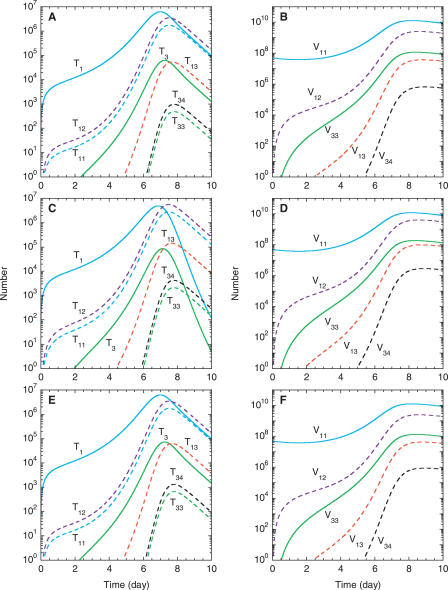
Model Predictions of the Dynamics of Different Infected Cell and Viral Subpopulations The time evolution of the various singly (solid lines) and doubly (dashed lines) infected cell (left panels) and homozygous (solid lines) and heterozygous (dashed lines) viral subpopulations (right panels) following the onset of infection. Note that *T*_1_ = *T*_2_, *T*_11_ = *T*_22_, *T*_3_ = *T*_4_, *T*_33_ = *T*_44_, *T*_13_ = *T*_23_ = *T*_14_ = *T*_24_, *V*_11_ = *V*_22_, *V*_33_ *= V*_44_, and *V*_13_ *= V*_23_ *= V*_14_ *= V*_24_. The parameter values employed are the same as those in Figure 2 except that *t*_d_ = 2.8 d in (C) and (D) and *ρ* = 10^−3^ crossovers per position in (E) and (F).

In Figure 3A, we present the distribution of the infected cells, T^*^, in Figure 2 into various singly and doubly infected cell subpopulations. We find that the various infected cell subpopulations also follow the two-phase dynamics above. The relative prevalence of the latter subpopulations is coupled to that of the corresponding viral subpopulations, which we present in Figure 3B. Because virions *V*_11_ and *V*_22_ alone are employed at the onset of infection, their numbers are larger than those of other viral subpopulations. When target cells are abundant, CD4 down-modulation ensures that singly infected cells occur more frequently than doubly infected cells. Thus, during the first phase of the dynamics following the onset of infection, cells singly infected with the infecting genomes, i.e., *T*_1_ and *T*_2_, are the most prevalent. Note that because *V*_11_ *= V*_22_ = *V*_0_ at the time of infection, at all subsequent times *T*_1_ = *T*_2_. Next in prevalence are cells infected twice with genome 1 and/or 2. Because coinfection by genomes 1 and 2 is twice as likely as double infection by either 1 or 2, cells *T*_12_ are more prevalent than *T*_11_ (= *T*_22_). The population of heterozygous virions, *V*_12_, increases because of viral production from the coinfected cells *T*_12_.

Infections by *V*_12_ give rise to cells *T*_3_ and *T*_4_, infected singly with the recombinant genomes, which in turn produce virions *V*_33_ and *V*_44_, respectively. Coinfection by genomes 1 and 3 yields cells *T*_13_, whose numbers are larger than those of the doubly infected cells *T*_33_ (= *T*_44_) because of the small population of *V*_33_ compared to *V*_11_. Again, because coinfection by genomes 3 and 4 is twice as likely as double infection by either 3 or 4, cells *T*_34_ are larger in number than *T*_33_. Yet, homozygous virions *V*_33_ are more prevalent than heterozygous virions *V*_34_ because cells *T*_3_, *T*_33_, and *T*_34_ produce *V*_33_, whereas cells *T*_34_ alone produce *V*_34_.

In the second dynamical phase, infected cell subpopulations decline because of cell death at rate *δ*. Singly infected cell subpopulations decline additionally because of second infections. Viral populations decline at the clearance rate *c*. The overall two-phase dynamics and the relative prevalence of various infected cell subpopulations are again in agreement with in vitro experiments [7].

Changes in the initial viral load, 2*V*_0_, or the infection rate, *k*_0_, do not alter the dynamics above qualitatively (unpublished data) [7,29]. Importantly, the CD4 down-modulation timescale, *t*_d_, does not influence the overall dynamics in Figure 2 (unpublished data). We assume here that viral production from cells is independent of the number of infections cells suffer, which is expected when viral production is limited by cellular rather than viral factors. Changes in *t*_d_ then alter the distribution of infected cells into various multiply infected cell subpopulations but do not alter the total population of infected cells, T^*^, or, consequently, the overall viral dynamics [29]. In Figure 3C and 3D, we present the calculations in Figure 3A and 3B with *t*_d_ = 2.8 d. A higher value of *t*_d_ implies slower CD4 down-modulation, which renders infected cells susceptible to further infections for longer durations and hence increases the relative prevalence of multiply infected cells. Accordingly, we find that doubly infected and coinfected cell subpopulations are higher in Figure 3C than in Figure 3A. (The faster decline of singly infected cells in the second phase in Figure 3C compared to that in Figure 3A is due to increased second infections in the former.) Correspondingly, the relative prevalence of heterozygous and recombinant virions increases upon increasing *t*_d_ (Figure 3B and 3D).

Interestingly, the recombination rate, *ρ*, also does not influence the dynamics in Figure 2 (unpublished data). We assume here that viral fitness is not affected by the mutations at the positions *l*_1_ and *l*_2_. Consequently, an increase in *ρ* increases the relative prevalence of recombinant genomes in the viral population but not the total viral load or the frequency of multiple infections. In Figure 3E and 3F, we present the calculations in Figure 3A and 3B with *ρ* = 10^−3^ crossovers per position. Note that the numbers of cells singly and doubly infected with genomes 1 and/or 2 are identical to those in Figure 3A, indicating that the frequency of multiple infections remains unaltered by the increase in *ρ*. The relative prevalence of cells infected with genomes 3 and 4, however, and that of the recombinant virions *V*_33_ (= *V*_44_) is higher in Figure 3E and 3F than in Figure 3A and 3B, respectively, because of the enhanced frequency of recombination in the former.

#### Scaling.

**Figure 4:**
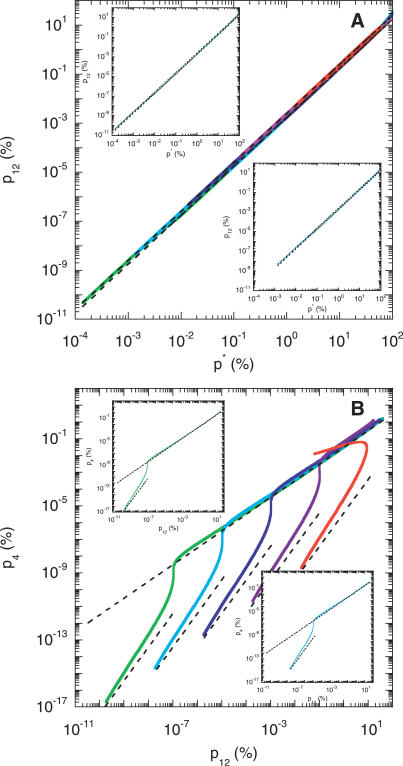
Model Predictions of Scaling Patterns Parametric plots of (A) the percentage of cells coinfected with genomes 1 and 2, *p*_12_, versus the total percentage of infected cells, p^*^, and (B) the percentage of cells infected with the recombinant 4, *p*_4_, versus *p*_12_, obtained by solving Equations 1–6 for different initial viral loads, 2*V*_0_ = 10^6^ (green), 10^7^ (cyan), 10^8^ (blue), 10^9^ (purple), and 10^10^ (red). The dashed lines are scaling patterns predicted by Equation 7 . The insets show the parametric plots for the individual cases, 2*V*_0_ = 10^6^ (green) and 10^7^ (cyan).

We examine next whether the above dynamics captures the scaling relationships between the different infected cell subpopulations observed experimentally [7]. In Figure 4A, we present parametric plots of the percentage of cells coinfected with genomes 1 and 2, *p*_12_ = 100*T*_12_ */*(T^*^ + *T*), versus the total percentage of infected cells, p^*^ = 100T^*^/(T^*^ + *T*), for different initial viral loads and with the parameter values employed in Figure 2. Remarkably, we find that for all the initial viral loads considered, *p*_12_ is proportional to (p^*^)^2^. The scaling behavior is observed over the entire period of infection (*t* = 10 d) including both the phases of the overall dynamics of Figure 2. Further, the parametric plots of *p*_12_ versus (p^*^)^2^ for different viral loads are superimposed, in agreement with the robust scaling observed in experiments [7].

In Figure 4B, we present the corresponding variation of the percentage of cells infected with the recombinant 4, 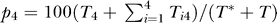 , with the percentage of coinfected cells, *p*_12_. Interestingly, we find two scaling regimes. When *p*_12_ is small, *p*_4_ is proportional to (*p*_12_)^2^, and the parametric plots are distinct for different values of *V*_0_. For larger values of *p*_12_, *p*_4_ is linearly proportional to *p*_12_ and independent of *V*_0_. Thus, the parametric plots in the latter regime are again superimposed, as observed in experiments [7].

We explain the origins of the above scaling regimes by considering two limiting scenarios in our model (Methods). First, for times small compared to the CD4 down-modulation timescale, i.e., *t ≪ t*_d_, and when changes in viral and cell numbers are small, we find that

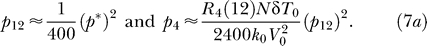
Second, for times large compared to the time required for viral load evolution to reach pseudo steady state, i.e., *t ≫ t*_eq_, we obtain

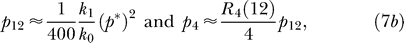
where we define *k*_1_ as the mean rate of the second infection of singly infected cells. As we show in Figure 4, Equation 7a and 7b capture the scaling regimes predicted by our simulations.

Remarkably, the scaling 
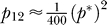
 (Equation 7a) is independent of model parameters and viral and cell numbers. Further, the quadratic scaling between *p*_12_ and p^*^ continues to hold for *t > t*_eq_ (Equation 7b ), with the proportionality constant lower than 1/400 by a factor *k*_1_ */k*_0_. We notice thus that a transition from the small time (*t ≪ t*_d_) scaling, 
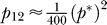
 , to the large time (*t* ≫ *t*_eq_) scaling, 
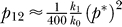
 , occurs in the parametric plots in Figure 4A. The transition occurs at larger values of p^*^ with increasing initial viral load. We find that a value of *k*_1_ = 1.4 × 10^−10^ d^−1^ captures the long-time scaling for all initial viral loads considered. On the other hand, the scaling between *p*_4_ and *p*_12_ for *t* ≪ *t*_d_, 
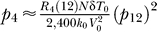
 , depends on model parameters and the initial viral load (Figure 4B). Interestingly, however, the linear scaling between *p*_4_ and *p*_12_, 
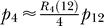
 , for *t > t*_eq_ is independent of the initial viral load.

For very large viral loads (2*V*_0_ ≥ 10^10^) and/or infection rates (*k*_0_ ≥ 2 × 10^−9^ d^−1^; unpublished data), we find that rapid infection and the consequent death of infected cells preempts the establishment of pseudo steady state between viral production and clearance in the first phase of infection, so that the linear scaling relationship between *p*_4_ and *p*_12_ is not observed (Figure 4B). Below, we compare model predictions with experiments.

### Comparison with Experiments

Available in vitro experiments, where cells are simultaneously exposed to two distinct kinds of viral genomes, may be segregated into two categories. First, single-round infection experiments employ replication-incompetent (heterozygous) virions to infect cells, and measure the fraction of cells that contain recombinant proviral genomes [3,7,13,14]. Second, viral dynamics experiments employ replication-competent (homozygous) virions and determine the time evolution of populations of cells infected with recombinant genomes [7]. We employ our description of the recombination probability (Equation 5b ) to predict data from single-round infection experiments and our entire model (Equations 1–6) to describe the latter viral dynamics experiments.

#### Single round of infection.

**Figure 5:**
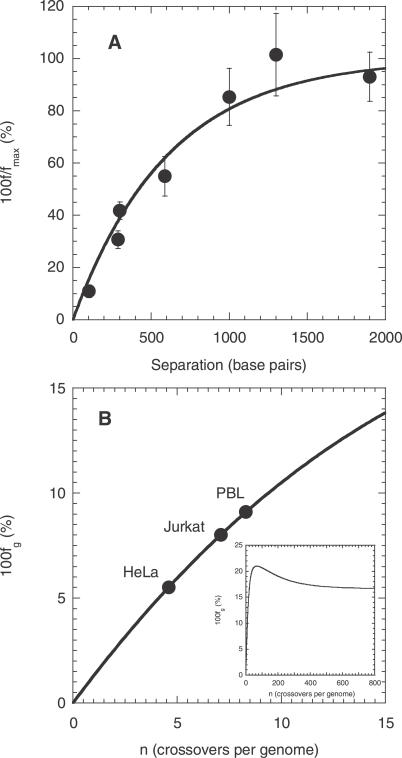
Comparisons of Model Predictions with Data from Single-Round Infection Experiments (A) The ratio of the percentage of cells infected with the recombinant 4, *f*, and the theoretical maximum percentage, *f*_max_, as a function of the separation, *l*, between the mutations on genomes 1 and 2 (see Figure 1) determined by Rhodes et al. [14] (circles) and by Equation 8 (line) with *ρ* = 8.3 × 10^−4^ crossovers per position. (B) The percentage of GFP^+^ cells as a function of the crossover frequency, *n*, determined by Equation 9 (line), on which are mapped the experimental percentages (circles) obtained by Levy et al. [7] with HeLa CD4, Jurkat, and primary T cells (PBL). The inset shows the prediction of Equation 9 over a larger range of values of *n*.

We consider single-round infection experiments, where target cells are exposed to a mixed viral population comprising homozygous virions, *V*_11_ and *V*_22_, and heterozygous virions, *V*_12_, in the proportions 1/4, 1/4, and 1/2, respectively. Small viral loads are employed so that multiple infections of cells are rare. Following infection, cells in which recombinant proviruses result are identified. Rhodes et al. [14] varied the separation *l* between the distinguishing mutations on genomes 1 and 2 (Figure 1A) and measured the fraction, *f*, of infected cells that contained the recombinant genome 4, which carries neither of the distinguishing mutations on genomes 1 and 2 (Figure 1B). Rhodes et al. report the latter fraction as a percentage of the theoretical maximum fraction, *f*_max_, attained at arbitrarily large separations and/or recombination rates (see below). We reproduce the experimental data of Rhodes et al. in Figure 5A.

We estimate the percentage of cells infected with genome 4 in the experiments of Rhodes et al. as follows. We recognize that cells infected with heterozygous virions *V*_12_ alone may possess the recombinant provirus 4. With the above distribution of the viral subpopulations, the probability that an infection is due to a heterozygous virion is 1/2. Following infection by a heterozygous virion, the probability that recombination yields genome 4 is *R*_4_(12) (Equation 5). Thus, the fraction, *f*, of infected cells that contain genome 4 is expected to be (1/2)*R*_4_(12) = (1/4)exp(−*ρl*)sinh(*ρl*). This fraction attains a maximum value, *f*_max_, of 1/8 (or 12.5%) as *ρl* → ∞. (When *ρl* → ∞, a large number of crossovers occurs between *l*_1_ and *l*_2_; the mutations at *l*_1_ and *l*_2_ are then selected independently, each with a probability 1/2, so that *R*_4_(12) → 1/4.) Thus, according to our model, *f*/*f*_max_ = [(1/2)*R*_4_(12)] / (1/8), which upon combining with Equation 5 yields

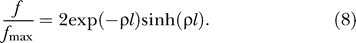



We fit predictions of Equation 8 to the experimental data of *f/f*_max_ versus *l* using *ρ* as an adjustable parameter (Figure 5A). Our model provides a good fit to the data, representing a successful test of our description of the recombination probabilities *R_i_*(*jh*) (Equation 5). The best-fit estimate of *ρ* = 8.3 × 10^−4^ crossovers per position indicates that *n* ≈ 8 crossovers occur on average (95% confidence interval: 6–10) in a genome of length *L* = 9,700 nucleotides. This estimate of *n* is in excellent agreement with a direct estimate from sequence analysis of ∼7.5 crossovers in a genome of 9,700 nucleotides [7]. We employ the best-fit estimate of *ρ* = 8.3 × 10^−4^ crossovers per position in our calculations above.

Levy et al. [7] also performed single-round infection experiments, where they exposed target cells simultaneously to homozygous reporter viruses containing either the cyan fluorescent protein (CFP) gene or the yellow fluorescent protein (YFP) gene, and heterozygous viruses with one strand containing the CFP gene and the other the YFP gene. The CFP and YFP genes were obtained by introducing specific mutations in the green fluorescent protein (GFP) gene. Thus, recombination events between the CFP and YFP genes that omit both the CFP and the YFP mutations yield the GFP gene. In addition, Levy et al. [30] observed that the CFP gene has certain distinguishing mutations between nucleotide positions ∼440 and ∼500. When recombination includes both the critical CFP and YFP mutations, and also the latter distinguishing mutations on the CFP gene, the resulting genome exhibits green fluorescence. When the latter mutations are not included, however, the resulting genomes remain undetected. Levy et al. determined the percentage of infected cells that exhibited green fluorescence as a measure of the recombination rate.

To compare the observations of Levy et al. with our model predictions, we let genome 1 (Figure 1A) represent the reporter virus with the CFP gene carrying the critical CFP mutation at *l*_1_ = 201 and genome 2 the virus with the YFP gene carrying the critical YFP mutation at *l*_2_ = 609, so that *l* = *l*_2_ − *l*_1_ = 408 [7]. We redefine genome 4 to encompass all genomes capable of green fluorescence. Thus, genome 4 includes genomes with the GFP gene, which contains neither of the mutations at *l*_1_ and *l*_2_, and also those genomes that contain both the mutations at *l*_1_ and *l*_2_ and the contents of genome 1 from positions 440 to 500. Accordingly, genome 3 includes those genomes that carry both the mutations at *l*_1_ and *l*_2_ but not all of the contents of genome 1 from positions 440 to 500. With these definitions of genomes 3 and 4, we recalculate the recombination probabilities of Equation 5d and find

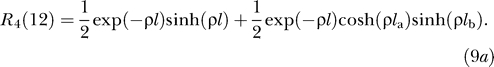
The first term on the right-hand side of Equation 9a is the probability that recombination excludes both the mutations at *l*_1_ and *l*_2_ and is given by Equation 5d. The second term represents the contribution to *R*_4_(12) that arises from recombination events that include both the mutations at *l*_1_ and *l*_2_ and the contents of genome 1 from positions 440 to 500. The latter contribution is determined as follows. The probability that reverse transcription begins on genome 1 at position *l*_1_ = 201 is 1/2. Given that the mutation at *l*_1_ is included, reverse transcriptase would be on genome 1 at position 440 if an even number of crossovers occurred between positions *l*_1_ and 440, which happens with the probability exp(−ρ*l*_a_)cosh(ρ*l*_a_), where *l*_a_ = 440 – *l*_1_. For the contents of genome 1 between positions 440 and 500 to be included in the resulting provirus, no crossovers must occur between positions 440 and 500, the probability of which is exp(−ρ60). Finally, the mutation at *l*_2_ = 609 on genome 2 is included if an odd number of crossovers occurs between positions 500 and *l*_2_, which happens with the probability exp(−ρ*l*_b_)sinh(ρ*l*_b_), where *l*_b_ = *l*_2_ – 500. Multiplying the latter probabilities and recognizing that *l*_a_ + *l*_b_ + 60 = *l* yields the second contribution to *R*_4_(12) above. Similarly, we find that

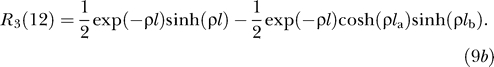



We determine the fraction of infected cells that fluoresce following exposure of cells to homozygous CFP and YFP virions and heterozygous CFP/YFP virions in the proportions 1/4, 1/4, and 1/2, respectively, as follows. (Fluorescent cells are detected in the experiments as infected.) When single infections of cells predominate, half of the infections are due to homozygous virions, which cause cells to fluoresce regardless of recombination. The other half of the infections, which are due to heterozygous virions, induce fluorescence when recombination yields genome 1, 2, or 4. Levy et al. ignore GFP^+^ cells in their estimate of the total fraction of infected cells [30]. The latter fraction is thus 1/2 + (1/2)(*R*_1_(12) + *R*_2_(12)). The experimentally determined fraction, *f*_g_, of infected cells that are GFP+ is therefore (1/2)*R*_4_(12)/[(1/2) + (1/2)(*R*_1_(12) + R2(12))], which simplifies to

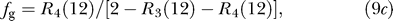
where *R*_3_(12) and *R*_4_(12) are determined using Equations 9a and 9b, respectively.

Levy et al. [7] report the mean percentage of infected cells that are GFP+ to be 8.0 with Jurkat T cells, 5.5 with HeLa CD4 cells, and 9.1 with primary CD4^+^ T cells. We compare these percentages with our prediction of *f*_g_ and estimate the recombination rate in the respective cell types (Figure 5B). We find that the mean number of crossovers in a genome of 9,700 nucleotides is 7.1 in Jurkat T cells, 4.6 in HeLa CD4 cells, and 8.3 in primary CD4^+^ T cells. Direct sequence analysis from Jurkat T cells showed a mean crossover frequency of 7.5 (range 3–13) [7], in excellent agreement with the estimate obtained here and from our analysis of the experiments of Rhodes et al. [14] above. Whereas the mean crossover frequency in HeLa cells is lower, that in primary CD4^+^ T cells is again in excellent agreement with the estimate for Jurkat T cells and that from the data of Rhodes et al. [14].

With macrophages, Levy et al. [7] found that ∼29% of infected cells are GFP^+^. We note that *f*_g_ defined in Equation 9c is a non-monotonic function of *ρ*: increasing *ρ* increases the probability of the accumulation of both the critical YFP and CFP mutations at *l*_1_ and *l*_2_ but lowers the probability that no crossovers occur between the positions 440 and 500 on genome 1. As a result, the second contribution to *R*_4_(12) in Equation 9a increases first and then decreases upon increasing *ρ*. Thus, upon increasing *ρ*, *f*_g_ increases (*f*_g_ = 0 when *ρ* = 0), reaches a maximum value of ∼21% at *ρ* = 0.007 crossovers per position (∼68 crossovers in 9,700 nucleotides), and declines to an asymptotic value of ∼16.7% as *ρ* → ∞ (Figure 5B, inset). The 29% GFP^+^ cells observed with macrophages is thus higher than the maximum value of *f*_g_ predicted by our model. We note that a higher percentage of GFP^+^ cells than the theoretical maximum of ∼21% may result if cells are multiply infected, which we ignore in our description of single-round infection experiments. Indeed, Levy et al. [30] observed that a large percentage of macrophages are coinfected despite the low viral loads employed. (Levy et al. reanalyzed their experiments [7] by accounting for double and triple infections of cells and estimated *ρ* [30]; the differences in their estimates of *ρ* and our estimates above may be attributed to the occurrence of multiple infections, which we ignore.) In contrast, Chen et al. and Rhodes et al. found no significant distinction between different cell types in their experiments [13,14]. Whether nonrandom infection processes [4,5] favored enhanced multiple infections of macrophages in the experiments of Levy et al. [7] remains to be ascertained.

#### Dynamics and scaling.

**Figure 6:**
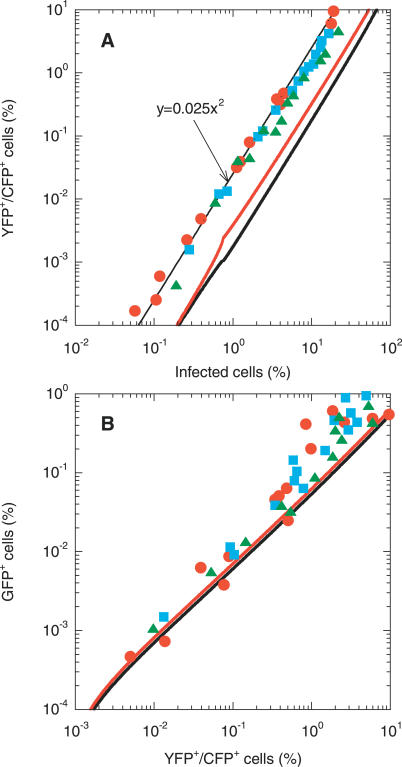
Comparisons of Model Predictions with Experimental Scaling Relationships Model predictions (thick lines) obtained by solving Equations 1–6, but withEquation 5d replaced by Equations 9a and 9b, compared with experimental scaling relationships (symbols) between (A) the percentage of coinfected cells (YFP^+^/CFP^+^) and the total percentage of infected cells, and (B) the percentage of GFP^+^ cells and the percentage of coinfected cells. The different symbols represent experiments conducted with cells from different donors [7]. Parameters employed for calculations are identical to those in Figure 2 except that for the red lines *t*_d_ = 2.8 d in (A) and *ρ* = 10^−3^ crossovers per position in (B). The thin black line in (A) is the experimental best-fit line [7].

We next compare our predictions with the dynamical and scaling patterns that Levy et al. [7] observed in their experiments with replication-competent viruses. Levy et al. employed equal populations of homozygous CFP and YFP reporter viruses to infect ∼10^6^ CD4^+^ T cells and detected the total percentage of cells infected (i.e., that fluoresced), p^*^, the percentage of cells that were coinfected with CFP and YFP genomes, *p*_12_, and the percentage of cells that were GFP^+^, *p*_4_, with time following the onset of infection. The quantities evolved in two distinct phases—an initial rise and a subsequent fall. Our model captures the two-phase dynamics qualitatively, as we demonstrate in Figure 4 (see “Model Predictions” above), and elucidates the origins of the two phases and of the observed relative prevalence of different infected cell subpopulations. Quantitative comparisons with the dynamical data are precluded by the possible presence in the experimental cultures of cells not susceptible to infection, which we discuss below. We focus here on the corresponding scaling relationships observed by Levy et al. [7]. In Figure 6A, we reproduce the experimental scaling relationship observed between *p*_12_ and p^*^, and in Figure 6B, the relationship between *p*_4_ and *p*_12_.

In Figure 6, we also present model predictions of *p*_12_ versus p^*^ and *p*_4_ versus *p*_12_ for the initial viral load 2*V*_0_ = 10^8^ and with the parameters employed in Figure 4. In the calculations in Figure 6, however, we replace Equation 5d for *R*_3_(12) and *R*_4_(12) by Equations 9a and 9b and ignore cells infected with genomes 3 alone, i.e., *T*_3_ and *T*_33_, in our count of the total number of infected cells, T^*^, because the latter cells do not fluoresce and remain undetected in the experiments [7]. We recognize that unlike in single-round infection experiments, the other recombination probabilities involving genome 4, *R_i_*(*j*4) in Equation 5 must also be altered to differentiate between the two kinds of GFP^+^ genomes (see above) present in the experiments. Based on the relative magnitudes of the two contributions to *R*_4_(12) in Equation 9a (for *ρ* = 8.3 × 10^−4^ crossovers per position, the values of the two terms are ∼0.12 and ∼0.03, respectively), we expect, however, that a majority of the GFP^+^ genomes are those that contain neither of the mutations on genomes 1 and 2, i.e., as shown in Figure 1B. We therefore employ, as an approximation, the remaining recombination probabilities as defined in Equation 5.

We find that our model captures the quadratic scaling, *p*_12_ ∼ (p^*^)^2^, qualitatively. Our model predicts that for small values of p^*^, the scaling relationship 
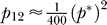
 holds (Equations 9a and 7a), and that it transitions to 
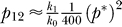
 for larger values of p^*^ (7b). Because *k*_1_ */k*_0_ < 1, the parametric plot of *p*_12_ versus p^*^ exhibits a parallel shift to lower values of *p*_12_ at large values of p^*^. Indeed, this shift is also observed in experiments, where the data lie on the experimental best-fit scaling relationship, 
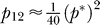
 , for small p^*^, but below the best-fit line for large p^*^.

Quantitatively, our model underpredicts the percentage of coinfected cells *p*_12_ compared to the experiments: the experimental proportionality constant relating *p*_12_ and p^*^, 1/40, is an order of magnitude larger than that estimated by our model, 1/400. One reason for this discrepancy might be the presence in the experimental cultures of cells not susceptible to infection. Hypothesize, for instance, the presence of a population, *T*_ns_, of non-susceptible cells in culture. The percentage of infected cells, p^*^, then becomes 100T^*^/(T^*^ + *T + T*_ns_), where *T* is now the susceptible target cell population governed by Equation 1, and T^*^ is the total population of infected cells. Similarly, the percentage of coinfected cells, *p*_12_, becomes 100*T*_12_ */*(T^*^ + *T + T*_ns_). The resulting proportionality constant, 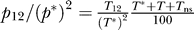 is greater than that determined by our model (*T*_ns_ = 0) by the factor 1 + *T*_ns_/(*T* ^*^ + *T*). An estimate of the latter factor is obtained by noting that the maximum percentage of cells infected in experiments is ∼20% for the two highest initial viral loads employed [7]. At the peak infection, we may assume that nearly all susceptible cells are infected, i.e., *T* ≈ 0, so that *T* ^*^/(*T* ^*^ + *T*_ns_) ≈ 1/5. Thus, the factor above, 1 + *T*_ns_/*T* ^*^ ≈ 5, explains at least in part the difference between the experimental proportionality constant and that derived from our model. Further, uncertainties exist in our knowledge of the CD4 down-modulation timescale, *t*_d_, of the cells in culture [26,28,29]. A larger value of *t*_d_ may enhance the frequency of multiple infections and increase *p*_12_ for a given value of p^*^. Indeed, our model predictions assuming *t*_d_ = 2.8 d appear to be in better agreement with the experimental scaling between *p*_12_ and p^*^ (Figure 6A). We note, however, that *t*_d_ = 2.8 d implies that *k*_1_ ≈ *k*_0_ throughout, so that several assumptions underlying the scaling relations in Equation 7 are not expected to hold. In particular, we find that for large p^*^, the proportionality constant relating *p*_12_ and p^*^ is higher than 1/400, the value of the constant for small p^*^, in contrast to that predicted for smaller values of *t*_d_ and observed in the experimental data. Nonetheless, quantitative comparison with the experimental scaling between *p*_12_ and p^*^ requires a description of the dynamics of the non-susceptible cell population including possible transitions from non-susceptibility to susceptibility and vice versa due to stimulation by regular IL-2 addition and loss of cell activation, respectively, which is beyond the scope of the present paper.

The presence of non-susceptible cells, however, does not influence the linear scaling relationship between *p*_12_ and *p*_4_. Given that 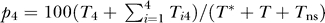 , the proportionality constant for the linear scaling, *p*_4_/*p*_12_ ≈ 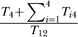 , is independent of *T*_ns_. Our model predicts that for small *p*_12_, *p*_4_ is proportional to (*p*_12_)^2^ and for large *p*_12_, *p*_4_ is proportional to *p*_12_ (Equation 7; Figure 4B). In the experiments, the quadratic scaling at small *p*_12_ is not observed [7]. For low initial viral loads, the transition from the quadratic to the linear scaling regime occurs at small values of *p*_12_ that may lie below experimental detection limits (Figure 4B). Upon increasing the initial viral load, the value of *p*_12_ at the transition increases but the quadratic scaling is short-lived. Thus, for larger viral loads, the transition to the linear scaling regime appears to occur before the first measurement following the onset of infection is made (at *t ≈* 2 d). Consequently, the linear scaling regime may alone be accessed in experiments.

We find remarkably that our model quantitatively captures the experimental linear scaling between *p*_4_ and *p*_12_ (Figure 6B). For small values of *p*_12_, the model is in excellent agreement with the data. Interestingly, the same recombination rate (*n* = 8 crossovers in a genome of 9,700 nucleotides) obtained from single-round infection experiments is employed in the latter predictions. (The latter predictions, however, are not adequately sensitive to changes in the recombination rate; calculations with a higher recombination rate, *ρ* = 0.001 crossovers per position [*n* = 10 crossovers in a genome of 9,700 nucleotides], yield only a marginal improvement in the comparison between model predictions and experiment [Figure 6B].) For large values of *p*_12_, the model slightly underpredicts the experimental data, possibly because of the increased likelihood of more than two infections of cells, which we ignore. Nonetheless, the quantitative agreement between model predictions and the experimental scaling relationship and the consistency of the predictions with the recombination rate estimated from independent single-round infection assays indicate that our model accurately captures the underlying dynamics of recombination during HIV infection.

## Discussion

The emergence of recombinant forms of HIV that are resistant to multiple drugs often underlies the failure of current antiretroviral therapies for HIV infection. Yet, the dynamics of the emergence of recombinant genomes in individuals infected with HIV remains poorly understood. Current models of HIV dynamics are unable to explain available experimental data of the frequency of occurrence and the time evolution of recombinant HIV genomes quantitatively. We developed a model that describes the dynamics of the emergence of recombinant forms of HIV and quantitatively captures key experimental observations. Mimicking recent experiments [5,7,14], we considered target cells exposed simultaneously to two kinds of homozygous virions. We constructed integral equations that predict the time evolution of the population of cells coinfected with both kinds of viruses. Following the first infection of a cell, viral gene expression induces CD4 down-modulation, which lowers the susceptibility of the cell to further infections. Because cells are infected asynchronously, determination of the frequency of multiple infections requires accounting for the different susceptibilities of individual cells to further infections at any given time based on the different times elapsed from their respective first infections, which is accomplished by the integral equation formalism [29]. Coinfected cells produce heterozygous progeny virions, which infect cells and yield recombinant proviral genomes. We developed a probabilistic description of template switching during reverse transcription and predicted the frequency with which heterozygous virions give rise to recombinant genomes. We integrated our descriptions of multiple infections of cells and recombination into standard models of HIV dynamics [15–17] and formulated dynamical equations that predict the time evolution of the populations of uninfected, singly infected, and multiply infected cells, and of homozygous, heterozygous, and recombinant viruses.

Model predictions are in agreement with the T cell dynamics observed in vitro. Levy et al. [7] found that following the onset of infection, the infected cell subpopulations evolve in two phases, an initial rise and a subsequent fall. Further, the percentage of cells infected by recombinant genomes, *p*_4_, is a small fraction of the percentage of coinfected cells, *p*_12_, which in turn is a small fraction of the total percentage of cells infected, p^*^. The two-phase dynamics and the relative prevalence of various infected cell subpopulations are in agreement with our model predictions. Our model also captures the scaling patterns relating the frequency of infection, coinfection, and recombination observed experimentally. Levy et al. [7] found remarkably that *p*_12_ is proportional to (p^*^)^2^ and that *p*_4_ is proportional to *p*_12_, independent of the initial viral load and the time following the onset of infection. Our model predicts both these scaling patterns and that the patterns are independent of the initial viral load and the time following the onset of infection. Quantitative comparison between our model predictions and the experimental scaling relationship between *p*_12_ and (p^*^)^2^ is precluded by the poorly characterized dynamics of cells not susceptible to infection by HIV that may be present in the experimental cultures. We showed, however, that the presence of non-susceptible cells does not influence the linear scaling relationship between *p*_4_ and *p*_12_. Indeed, our model predictions are in quantitative agreement with the experimental scaling relationship between *p*_4_ and *p*_12_. The quantitative agreement indicates that our model captures the underlying dynamics of HIV recombination accurately.

Our model also captures data from single-round infection experiments on the frequency of the accumulation by recombination of distinct mutations present on the two RNA strands within a virion. From comparisons of model predictions with the experiments of Rhodes et al. [14], we estimate that ∼8 template switches, or crossovers, occur on average (95% confidence interval: 6–10) during the reverse transcription of an entire HIV genome of ∼10^4^ nucleotides. This number is in agreement with independent estimates from direct sequence analysis by Levy et al. [7], who observed ∼7.5 crossovers (range 3–13) on average. Comparison of our model predictions with the single-round infection assays performed by Levy et al. yields crossover frequencies of ∼7.1 in Jurkat T cells, ∼4.6 in HeLa CD4 cells, and ∼8.3 in primary CD4^+^ T cells. Whereas the crossover frequency in HeLa cells is lower, the frequency in the other two cell types is in agreement with the estimate obtained from our analysis of the experiments of Rhodes et al. [14]. Further, the scaling relationship between *p*_4_ and *p*_12_ described above is also consistent with a recombination rate of ∼8 crossovers per ∼10^4^ nucleotides.

The power law scaling that the number of doubly infected cells is proportional to the square of the total number of infected cells is also predicted by the model of HIV dynamics with multiple infections developed by Dixit and Perelson [29]. In contrast to experiments, however, the predicted scaling is dependent on the time following the onset of infection and the initial viral load. Here, by considering percentages rather than numbers of infected cells and by distinguishing between cells doubly infected by a single kind of genome and coinfected with distinct genomes, we mimic experimental quantities more accurately and find that the scaling relationship is independent of the time following infection or the initial viral load, as observed in experiments [7]. Fraser argues that the quadratic scaling observed between the percentage of doubly infected cells and the total percentage of infected cells, the latter predominantly singly infected, may imply a deviation from mass action kinetics for the second (and perhaps further) infections of cells and suggests, motivated by the scaling, that the rate of second infection of cells may be proportional to the square of the viral load (*r* ∼ *kV* ^2^) [22]. Here, we find that the quadratic scaling emerges without deviations from mass action kinetics (*r* ∼ *kV*). Further, to address possible differences between the rates of first, second, and third infections, due, for instance, to CD4 down-modulation, Fraser postulates the use of different values of the rate constants, *k*, for successive infections. In our model, the differences in the rate constants for multiple infections follow naturally from our description of CD4 down-modulation (Equation 3). The latter description facilitates accurate estimation of the frequency of multiple infections under varying viral loads: when the viral load is high, for instance, the second infection of a cell may occur rapidly after its first infection, in which case the rate constants for the first and second infections are expected to be similar due to negligible CD4 down-modulation in the intervening interval. A fixed rate constant for second infection (independent of the viral load) would then tend to underestimate the frequency of double infections. The dependence on the viral load of the variation of the apparent infection rate constant with the number of infections implies that when viral load changes are rapid, the likelihood of a cell suffering multiple infections would depend on the instant of its first infection. For instance, in patients undergoing efficacious antiretroviral therapy, a cell first infected at the start of therapy, when the viral load is large, is expected to have a higher rate constant for second infection than a cell that is first infected a day after the onset of therapy, when the viral load is significantly reduced. Our integral equation formalism, which accounts for the asynchronous first infections of cells, allows accurate determination of the frequency of multiple infections and consequently the influence of recombination throughout the infection period.

If the distinction between different viral genomes is ignored, our model reduces to the model of HIV dynamics with multiple infections developed by Dixit and Perelson [29] when more than two infections of cells are rare. Indeed, changes in the total viral load, target cell numbers, and total infected cell numbers predicted by our model (Figure 2) are identical to those predicted by the latter model. Importantly, the latter model reduces to the standard model of HIV dynamics [15–17] when viral production from cells is independent of the number of infections cells suffer. Our model is thus consistent with the standard model of HIV dynamics, which successfully predicts viral load changes in patients following the onset of antiretroviral therapy [15–17]. Further, infections in SCID-hu mice also show scaling patterns similar to those observed in vitro [7] and predicted by our model, reinforcing the notion that our model may be applied to describe the dynamics of recombination in vivo.

Several advances of our model are essential, however, to predict the emergence of recombinant genomes in vivo. First, that infected splenocytes from two HIV patients harbored 3–4 proviruses per cell on average [6] suggests that multiple infections of cells may be more prevalent in vivo than is assumed in our model. Second, multiple infections in vivo may be orchestrated by cell–cell transmission as well as by free virions [32,33]. Third, HIV has a high mutation rate [34], which introduces genomic variations in vivo that may subsequently be accumulated by recombination. Indeed, the high mutation and turnover rates of HIV in vivo [17,35] suggest that the likelihood of the preexistence of individual drug resistance mutations in patients is high [36]: approximately half of the HIV patients in the United States are estimated to be infected with genomes that possess resistance to at least one of the currently available drugs [37]. Our model describes how preexisting mutations may become associated by recombination. Determination of the existence of individual mutations, however, requires a description of the HIV mutation process, which we ignore. Fourth, fitness interactions between mutations [24] modulate the relative prevalence of recombinant genomes, whereas we assume all viral genomes to be equally fit. We note that incorporating fitness selection enables our present model to describe additionally in vitro serial passage experiments of the emergence of drug resistance via recombination [2,38]. Finally, whereas we consider two loci, a description of recombination between more than two loci is essential in vivo, as more than two mutations are typically responsible for resistance to individual drugs [37]. With the above advances, some of which are suggested in currently available models [18,20,22,23], our model may facilitate prediction of the emergence of multi-drug–resistant strains of HIV in infected individuals.

## Methods

### Solution of dynamical equations.

We non-dimensionalize Equations 1–4 and 6 using the following dimensionless quantities:

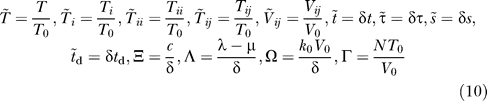
and obtain

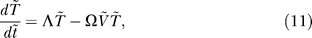


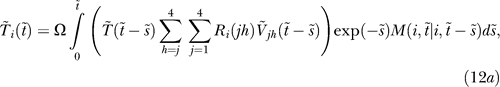


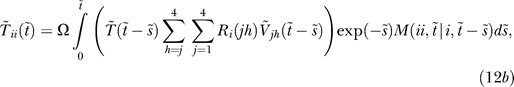


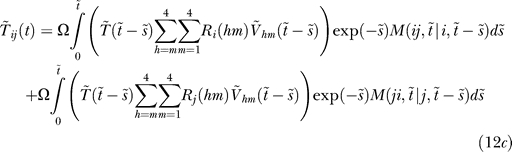


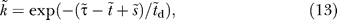


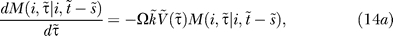


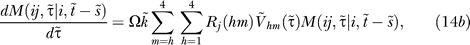


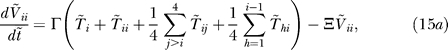
and

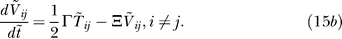



We solve the dimensionless Equations 5 and 11–Equation 15 as follows. We recognize that the equations are strongly coupled because of the integral equation formalism (Equation 12) employed; for instance, evaluation of the integral in Equation 12a to determine 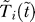 requires knowledge of 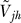 at all times from 0 to 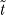 , which in turn depends on 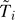 through Equation 15. Using the initial conditions, we first integrate the differential equations for 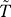 and 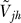 (Equations 11 and 15) for a small time step *θ*, i.e., from 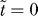 to 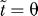 . Next, we integrate the differential equations for the conditional probabilities *M* (Equation 14) by discretizing 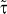 , which can vary from 0 to *θ*, into intervals of length *θ_m_* and determining 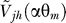 , where *α* assumes integer values from 0 to *θ/θ_m_*, by linear interpolation between 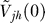 and 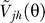 . We then evaluate the integrals in Equation 12 to determine 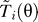 , 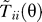 , and 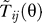 . We march forward in time and evaluate 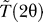 and 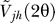 by integrating Equation1 11 and 15 from time 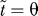 to 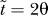 , integrate Equation 14 by allowing 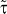 to vary from 0 to 2*θ*, and evaluate the integrals in Equation 12 to determine 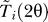 , 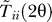 , and 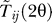 . We repeat the procedure until 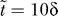 (i.e., *t* = 10 d). The solution is implemented by a computer program written in Fortran 90.

### Scaling analysis.

We derive below the scaling relationships mentioned in Equation 7. Following the onset of infection, for times small compared to the timescale of CD4 down-modulation, i.e., *t ≪ t*_d_, because *k* ≈ *k*_0_, we write the dynamics of singly infected cells as

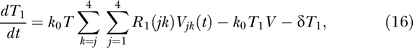
where the first term on the right-hand side is the rate of formation of *T*_1_ by the infection of uninfected cells, and the second and third terms are the losses of *T*_1_ due to further infections and cell death. At the start of infection, the dominant viral populations are *V*_11_ and *V*_22_ (Figure 3B), of which infection by the former alone yields *T*_1_. Further, because *T*_1_ is small (Figure 3A), the loss terms, which are linear in *T*_1_, are negligible. Equation 16 then simplifies to

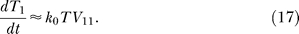
For lengths of time short compared to the timescales over which *V* and *T* change, we let *T* ≈ *T*_0_ and *V*_11_ ≈ *V*_0_ and integrate Equation 17 to obtain

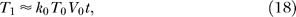
where we use the initial condition *T*_1_(0) = 0. With assumptions similar to those employed in obtaining Equation 17, we find that the coinfected cell population evolves according to the following equation:

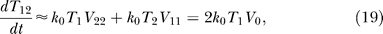
where we recognize that *T*_1_ = *T*_2_ and *V*_11_ = *V*_22_ ≈ *V*_0_. Substituting for *T*_1_ from Equation 18 and integrating with the initial condition *T*_12_(0) = 0, we get

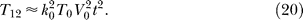
The evolution of the heterozygous viral population, *V*_12_, is given by

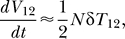
where we note that 1/2 of the virions produced from cells *T*_12_ are heterozygous. We ignore viral clearance because *V*_12_ is expected to be small. Substituting for *T*_12_ from Equation 20 and integrating with the initial condition *V*_12_(0) = 0, we obtain

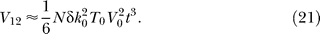
The time evolution of the cell population infected with recombinants, *T*_4_, is then given by

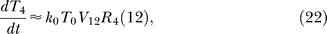
where we recognize that because *V*_12_ ≫ *V*_44_ ≫ *V*_14_, most of the cells *T*_4_ are formed due to infection by *V*_12_ followed by recombination. Substituting for *V*_12_ from Equation 21, and integrating with the initial condition that *T*_4_(0) = 0, we find

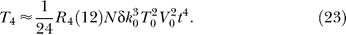



Because the total infected cell population comprises largely cells *T*_1_ and *T*_2_, which in turn are significantly smaller in number than uninfected cells (Figures 2 and 3A), we obtain the total percentage of infected cells,

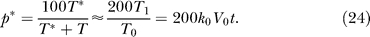
Similarly, the percentage of coinfected cells,

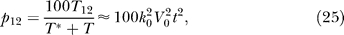
and the percentage of cells infected with recombinants,

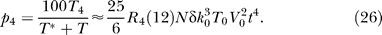
Combining Equations 24–26, we obtain

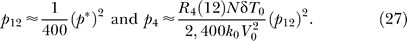
We thus find that early during infection, the scaling laws *p*_12_ ∼ (p^*^)^2^ and *p*_4_ ∼ (*p*_12_)^2^ hold.

We next consider times longer than the timescale over which viral production and clearance reach pseudo steady state, *t > t*_eq_. The magnitudes of the viral subpopulations still follow *V*_11_ = *V*_22_ ≫ *V*_12_ ≫ *V*_44_ (Figure 3B). Similarly, for the infected cell subpopulations, we have *T*_1_ = *T*_2_ ≫ *T*_12_ ≫ *T*_4_ (Figure 3A). The relevant evolution equations may then be written as

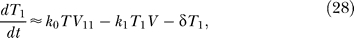


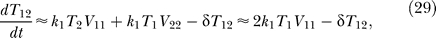


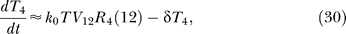


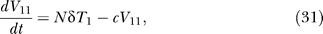
and

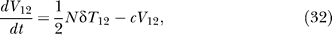
where we let *k*_1_ be the “mean” infection rate of singly infected cells. We note that *k*_1_ is a function of the CD4 down-modulation timescale, *t*_d_. If *t*_d_ is large, for instance, then *k*_1_ ≈ *k*_0_. Applying the pseudo steady state approximation for the viral populations yields

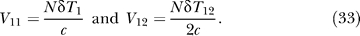
Substituting for *V*_11_ from Equation 33 in Equation 28, we obtain

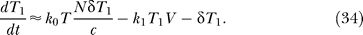
Assuming that changes in the target cell population, *T*, and the total viral load, *V*, occur slowly compared to changes in *T*_1_, which is expected in the initial stages of infection, we integrate Equation 34 to obtain

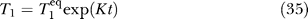
where 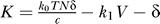 and 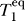 is the value of *T*_1_ when *t* = *t*_eq_. Note that in Equation 35, *t* ≫ *t*_eq_. Substituting for *V*_11_ and *T*_1_ in Equation 29 yields

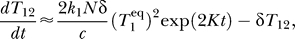
which upon integrating with the initial condition 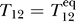 when *t = t*_eq_ and recognizing that 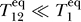 gives

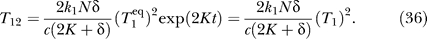
Combining Equations 30, 33, and 36, we get

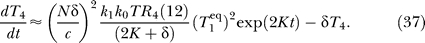
Integrating Equations 37 and assuming 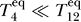 yields

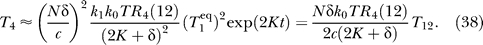
Again, assuming that the singly infected cells are predominant in the infected cell population, the percentage of total infected cells is

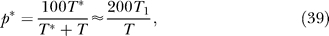
where the small percentage of infected cells allows us to write T^*^ + *T* ≈ *T*. The percentage of coinfected cells is then

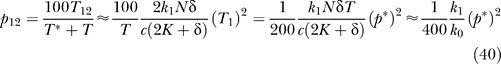
where the last approximation follows from the sharp rise in *T*_1_ following the establishment of pseudo steady state (Figure 3A), which according to Equations 34 implies that 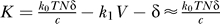 . Finally, the percentage of cells *T*_4_ infected with recombinants is

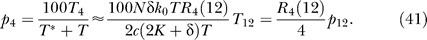
Thus, later in the infection (*t > t*_eq_), the scaling laws *p*_12_ ∼ (p^*^)^2^ and *p*_4_ ∼ *p*_12_ hold.

## Abbreviations

CFP: cyan fluorescent protein
GFP: green fluorescent protein
YFP: yellow fluorescent protein
